# Perspectives of Healthcare Providers to Inform the Design of an AI-Enhanced Social Robot in the Pediatric Emergency Department

**DOI:** 10.3390/children10091511

**Published:** 2023-09-06

**Authors:** Summer Hudson, Fareha Nishat, Jennifer Stinson, Sasha Litwin, Frauke Zeller, Brittany Wiles, Mary Ellen Foster, Samina Ali

**Affiliations:** 1 Department of Pediatrics, Faculty of Medicine and Dentistry, University of Alberta, Edmonton, AB T6G 1C9, Canada; kavan@ualberta.ca (S.H.); sali@ualberta.ca (S.A.); 2 Child Health Evaluative Sciences, The Hospital for Sick Children, Toronto, ON M5G 0A4, Canada; fareha.nishat@sickkids.ca (F.N.); brittany.wiles@sickkids.ca (B.W.); 3 Lawrence S. Bloomberg, Faculty of Nursing, University of Toronto, Toronto, ON M5T 1P8, Canada; 4 Division of Emergency Medicine, The Hospital for Sick Children, Toronto, ON M5G 1E8, Canada; sasha.litwin@sickkids.ca; 5 School of Computing, Engineering, and The Built Environment, Edinburgh Napier University, Edinburgh EH11 4BN, UK; f.zeller@napier.ac.uk; 6 School of Computing Science, University of Glasgow, Glasgow G12 8RZ, UK; maryellen.foster@glasgow.ac.uk

**Keywords:** artificial intelligence, social robotics, needs assessment, procedural distress, children, pain, co-design

## Abstract

Children commonly experience pain and distress in healthcare settings related to medical procedures such as blood tests and intravenous insertions (IVIs). Inadequately addressed pain and distress can result in both short- and long-term negative consequences. The use of socially assistive robotics (SARs) to reduce procedure-related distress and pain in children’s healthcare settings has shown promise; however, the current options lack autonomous adaptability. This study presents a descriptive qualitative needs assessment of healthcare providers (HCPs) in two Canadian pediatric emergency departments (ED) to inform the design an artificial intelligence (AI)-enhanced social robot to be used as a distraction tool in the ED to facilitate IVIs. Semi-structured virtual individual and focus group interviews were conducted with eleven HCPs. Four main themes were identified: (1) common challenges during IVIs (i.e., child distress and resource limitations), (2) available tools for pain and distress management during IVIs (i.e., pharmacological and non-pharmacological), (3) response to SAR appearance and functionality (i.e., personalized emotional support, adaptive distraction based on child’s preferences, and positive reinforcement), and (4) anticipated benefits and challenges of SAR in the ED (i.e., ensuring developmentally appropriate interactions and space limitations). HCPs perceive AI-enhanced social robots as a promising tool for distraction during IVIs in the ED.

## 1. Introduction

Children commonly experience pain and distress in healthcare settings, which is often associated with the required diagnostic and therapeutic procedures, such as blood tests and intravenous insertions (IVIs). Inadequately addressed pain and distress can result in negative consequences, both in the short-term (e.g., fear, prolonged/repeat procedures and impediment of care) and the long-term (e.g., needle phobia and health-care anxiety/avoidance) [1,2,3]. This highlights the need to develop methods to mitigate procedure-related pain and distress among children, as this is crucial for the delivery of quality and sufficient health care. Current methods include distraction therapies, such as bubble blowing, video games, music therapy, and virtual reality; these have been clinically shown to reduce children’s procedure-related pain and distress [4,5,6]. One technological avenue which has received less attention in pediatric settings is the use of socially assistive robotics (SARs). 

SARs is an area of robotics where the goal is for a robot to use speech, facial expressions, and gestures to interact with a human partner for the purpose of providing assistance [7]. SARs have been deployed successfully in a wide range of healthcare domains, particularly in the care for the elderly [8]. More recently, they have been applied in pediatric settings to assist with autism diagnosis and therapy [9,10] and to help children with pain and distress during short- and long-term hospitalizations [11,12,13]. However, a recent scoping review of social robotics in children identified important gaps in this area: small sample sizes, lack of end-users in the design process, non-clinical trial designs, and lack of effectiveness outcomes [11]. 

The utility of social robotics within the pediatric emergency department (ED) for distraction during brief needle-related procedures has recently been examined [14,15]. The initial results have been promising, demonstrating high acceptance among children and efficacy in reducing procedure-related distress. However, the existing studies are all hindered by a critical technical limitation: all robots were operated remotely and employed entirely scripted behaviour with very limited real-time responsiveness and a complete lack of autonomy. This limitation diminishes the robot’s flexibility to provide adaptive and personalized procedural support to children. Artificial intelligence (AI), the ability for computer systems to make autonomous decisions and independently select appropriate behaviours, has the potential to address these limitations, thereby yielding a more effective distraction tool. As such, we propose the design of an AI-enhanced SAR for use in the pediatric ED, with the specific aim of providing adaptive and flexible emotional support to reduce IVI-associated pain and distress.

Of the aforementioned previous works, only two SARs [Arash and Maya] developed for pediatric clinical settings have been reported and their design process described; these studies only included child participants [12,13]. We posit that more effective SARs could be created by ensuring that healthcare experts, engineers, and end users collaborate throughout the design process. One such process is the user-centered co-design methodology. This is an increasingly recognized technique in the design of interactive AI-enhanced systems in general [16] and is particularly applicable to the design of a SAR intended to interact with users in a specific real-world social setting. For such a robot, gathering the needs and desires of the target users is a crucial part of the design process. Co-design has been successfully used for the design of an SAR intended for deployment in real-world contexts, especially for vulnerable target populations. Some examples of this include an SAR designed to support children in creative processes [17], adolescent mental health [18], older adults with depression [19], robots fostering anti-bullying peer support [20], and robots for shopping mall wayfinding [21]. In many cases, the co-design process involves a discussion of both the form and the behaviour of the target robot [16,18]; however, even when the SAR form is fixed—as in the current project—the co-design process is still important to provide significant and valuable insights into the desired behaviours [21].

Our overall project goal is to develop an artificial intelligence (AI)-enhanced SAR for children’s healthcare, specifically targeting interactions that take place during IVIs in the ED. We aim to reduce children’s procedure-related pain and distress. The target robot platform is the humanoid Nao robot (SoftBank Robotics), which has been widely used in child–robot interaction experiments, including in several previous clinical studies of social robots [14,15]. The details of the overall project methodology are available in a previously published manuscript [22]. As part of the process of developing the behaviour of this system (both the repertoire of SAR behaviours as well as guidelines regarding when one behaviour should be selected over another), this study aimed to characterize healthcare practitioners’ (HCPs) (a) experience managing children’s procedural pain and distress, both generally and during IVI procedures, as well as (b) their perspectives surrounding the integration of technology, more specifically AI and robots, within the pediatric ED setting. The perspectives of children and their caregivers on these matters are characterized in a previous work [23].

## 2. Materials and Methods

We carried out a co-design study involving target stakeholders using a descriptive qualitative design as outlined by Sandelowski [24]. A purposive sample was used to maximize the variety of professional roles (physicians, nurses, and child life specialists) in the ED. Eligible HCPs were required to work in the pediatric ED at one of two tertiary care Canadian pediatric hospitals (The Hospital for Sick Children [Toronto, ON, Canada] and Stollery Children’s Hospital [Edmonton, AB, Canada]), be working in the ED for at least one year, and be able to be interviewed in English. Trainees were excluded from this study. Study information emails were distributed to pediatric ED physicians, nurses, and child life specialists at each institution. Interested HCPs contacted research staff via email, were provided with more information about the study, and underwent informed consent through Research Electronic Data Capture (REDCap), a secure online data collection platform [25]. All HCPs provided their demographic characteristics and technological experience through an online questionnaire via REDCap. Individual and group interviews were conducted with HCPs by two interviewers (SH and FN) between March and November 2021 virtually via Zoom and were audio-recorded. Semi-structured interview guides were created using the team’s expertise in conducting previous needs assessments [26,27,28] and a review of the existing literature. Interview questions were designed to capture [1] HCPs experience during IVI (e.g., challenges, available resources, and strategies), [2] perception of AI and robot technology, and [3] feedback based on previous use of social robotics in this healthcare context. Interviewers also used prompts to gain clarity from participant responses as needed. Institutional ethics approval was received from the SickKids Research Ethics Board [1000072883] and the University of Alberta Research Ethics Office [Pro00097697].

It is important to note that there are ethical issues that need to be considered when designing SARs. To do so, our team employed a vertically integrated ethics perspective. This allowed us to prioritize ethical inquiry throughout the project, from study conception to data collection and analysis for each phase of the project. Specific to the co-design, our interview guide included questions that would garner ethical perspectives from HCPs, touching on elements such as trustworthiness, consent of use, and safety. 

Audio recordings of interviews were transcribed verbatim. A content analysis approach was employed [24,29]. Interviews and focus groups were coded independently in Dedoose (Version 9.0.46), a cross-platform, cloud-based application for analyzing qualitative and mixed-methods data, by two team members (FN and BW). Initially, a subset of transcripts was reviewed by team members to develop a coding scheme (SH and FN) through an inductive approach. Subsequently, each transcript was coded twice by two individual team members (FN and BW). The coding scheme was iteratively revised as needed during this process until no new data could be categorized. Once coding was complete, overall content areas were extrapolated through a process of quotation review, which was finalized by two team members (SH and FN) and supervised by a senior team member (JS) with expertise in qualitative research and co-design.

## 3. Results

Eleven HCPs were recruited across both sites. At the Hospital for Sick Children, six HCPs completed individual interviews with the research team, and at Stollery Children’s Hospital, one focus group with five HCPs was completed. A multidisciplinary group of HCPs was recruited, including nurses, physicians, and child life specialists. Individual interviews lasted 40–60 min each, while the focus group lasted 80 min. 

As shown in the table below, the interviewed HCPs were largely female and had varying years of experience in healthcare (ranging from 2 to 41 years) and pediatric emergency medicine specifically (1–21 years). Some had experience with voice assistants, smart home technology, and robot technology (Table 1). 

Responses from semi-structured individual and group interviews were categorized into four major themes: (1) common challenges during IVI, (2) available tools for pain and distress management during IVI, (3) response to SAR appearance and functionality, and (4) anticipated benefits and challenges of SAR in the ED. 

### 3.1. Common Challenges during IVI

All HCPs agreed that managing pain and distress in the ED was important, and the time spent addressing these concerns could result in an overall better experience for the child and their family during the ED visit and positively impact future visits. HCPs experience common challenges while performing the IVI procedure. First, HCPs mentioned that managing the pain associated with the IVI was easier than managing the anxiety and distress many children experience. This anxiety or overall distress associated with the needle procedure can become so overwhelming for children that they are unable to distract themselves on their own or with typically available tools (i.e., smart phones and conversation).

“*Well, usually the emotion is beyond distractible. So if they are in too much pain or anxious, they’re [the child] kind of lost in that emotion at that point, you can either continue and just power through, or you have to kind of take a break, then pause, and let them collect themselves before proceeding*.”

—[Participant 8, Physician]

Furthermore, the approach used for children needs to be developmentally appropriate, specifically around the language used (e.g., ‘poke’ versus ‘needle’ for a younger child) to describe the IVI procedure. Child anxiety/distress can also be further exacerbated by parent anxiety/distress.

“*I find as soon as the caregiver is kind of doubting you, then that just escalates any anxiety in the room, and then it’s much more challenging*.”

—[Participant 9, Nurse]

Children also manifest distress in different ways than adults; they may squirm, shake their arms, or hide from HCPs, which can make performing the IVI difficult.

“…*getting them [the child] to hold still, whether it’s the parent holding them still and then you have to have faith that they can hold them, so you don’t miss if they flinch*.”

—[Participant 2, Nurse]

Further, given the urgent nature of some of the conditions that are present in the ED, HCPs may have to perform the IVI procedure without the availability of time to provide all the available pain and comfort measures.

“*Because we’re in a paediatric emergency department, there are issues of urgency. So having time constraints can direct how you’re going to approach pain*.”

—[Participant 4, Physician]

### 3.2. Available Tools for Pain and Distress Management during IVI

Pain during IVI can be managed by pharmacological and non-pharmacological tools. In a pediatric setting, HCPs often use topical creams (e.g., Maxilene) or vapocoolant sprays (e.g., Pain-Ease) as the primary pharmacological tool to numb the skin prior to an IVI. On occasion, a highly anxious child may require other medications for sedation or relaxation.

“*We always start with medical interventions, so either freezing spray or like a pain gel or sucrose on babies*.”

—[Participant 6, Nurse]

Non-pharmacological tools are particularly useful for distress management, with commonly used tools including watching videos, blowing bubbles, playing with small toys (e.g., fidget spinners and stress balls), deep breathing exercises (via apps or led by child life specialists), and engaging in distracting conversation (e.g., about favourite tv shows or sports teams). Technological tools are becoming increasingly available, including iPads and video games. Some HCPs also have experience with virtual reality headsets [28].

“*Utilizing iPad, we used to be able to use bubbles, which was a huge thing before COVID…And then light spinners, musical toys, even parents that just have a phone with YouTube or music on it is helpful*.”

—[Participant 7, Child Life Specialist]

### 3.3. Response to SAR Appearance and Functionality

All HCPs were shown a video of the Nao robot in a pediatric clinical setting, without AI-enhancement. Most HCPs liked the physical appearance of the robot; they found the colour, overall design, and size of the robot appropriate for a pediatric setting.

“*I think a kid would think it’s very, very cool…I think for a kid I don’t think it needs to be changed or perfected it’s probably going to work just fine*.”

—[Participant 6, Nurse]

Furthermore, they found the robot made the atmosphere more relaxed and would be a good distraction as a novel stimulus for most children.

“*I think the older kids definitely, and even some of the younger ones are going to love it because ‘what is this thing, this is pretty cool.’ So it’s going to keep them distracted*.”

—[Participant 11, Child Life Specialist]

HCPs also found the behaviour and movement used by the exemplar robot helpful and appropriate. The HCPs suggested that the robot have the ability to adapt and target its behaviour, actions, and speech to the developmental age of the child. For example, although many HCPs found the language the robot used helpful, the presented exemplar interaction felt most appropriate for young children.

“*I think it has to depend on the child’s age…and then as they get to be older like that child’s age [referring to the patient in the video], I think explaining everything and then also maybe doing some distraction in between…and then as the child gets older, definitely explaining what’s going to happen and then reading the child and walking through the deep breathing or even engaging in a conversation like we would do for these kids like, ‘Oh, do you like to snowboard? Tell me about snowboarding.’ It definitely depends on the age of the child*.”

—[Participant 9, Nurse]

While the above quote suggests the explanation of the IVI procedure by the robot to be helpful, overall opinions from the HCPs were mixed, with most HCPs preferring that the staff explain the IVI to the child rather than the robot. HCPs also suggested that older children should have distraction options to choose from.

“*I think that’s like totally up to the child...Do you want me [in reference to the robot] to dance? Do you want me to sing? Let me read you a story...maybe some options and then they choose and that’s what it does*”

—[Participant 7, Child Life Specialist]

Moreover, HCPs found the robot passive and wished for more dynamic behaviour from the robot, which would be more engaging to the child.

“…*being a little bit more animated physically and a level of talking that is more consistent with the age group that it’s aimed at.*”

—[Participant 4, Physician]

### 3.4. Anticipated Benefits and Challenges of SAR in the ED

HCPs noted both potential benefits and challenges to incorporating an AI-enhanced SAR into the ED for IVI procedures. The benefits largely focused on the role the robot may play before, during, and after the IVI procedure. One HCP described the usefulness that both the physical presence of the robot and the social responsiveness of the AI-enhancement may bring.

“*I kind of see it in a similar role as [the] child-life [specialist] being able to have their sole focus on the child and interacting with them while we worry about the not-so-fun stuff. If they’re [referring to the AI-enhanced SAR] able to pick up and read off their emotions, I could see that as being super helpful and then you don’t direct the robot at all, if they’re able to see that the child is scared, and then change their approach based on that. I could see that being very helpful, I could see even having a robot come into a lot of kids’ rooms and just that alone being enough to be like, ‘I don’t care what you’re doing, do whatever you want with my hand like I just want to play with this.’ I could see that being very helpful for distraction and changing the whole atmosphere of the room*.”

—[Participant 9, Nurse]

Before the procedure, HCPs suggested that the SAR could assist by reducing the child’s anxiety through cognitive behavioural techniques such as breathing exercises, guided imagery, or meditation. HCPs highlighted the importance of practicing these coping skills and, as such, suggested the robot be present in the room prior to the clinical team’s arrival for the IVI.

“*If there was something where they could just close their eyes and either it be reading a story or pretending, they’re on a beach and listening to waves and you can hear the waves in the background…Getting the child engaged before the actual procedure would be good, because if you’re if you’re just bringing it [in right before]…there’s a lot happening at once*.”

—[Participant 7, Child Life Specialist]

Additionally, making the child comfortable through humour was also thought to be beneficial.

During the procedure, HCPs saw the primary role of the SAR as distracting and engaging the child since pain is generally well addressed using pharmacological tools. This distraction could include playing a video, singing a song, reading a book, or playing a game (e.g., Simon Says). Additionally, encouraging the child by providing positive affirmations such as “*you’re doing a really good job, you’re doing so well*” was also felt to be of value. Finally, HCPs believe the SAR may also have a significant impact after the procedure is complete, as most clinical staff move on promptly to the next patient. During this period, the SAR could provide positive reinforcement.

“*Positive reinforcement is good because they all want to do a good job. They [the patients] try very hard to do a good job, but we ask a lot of them*.”

—[Participant 11, Child Life Specialist]

Several HCPs also mentioned the importance of reminding children that they, themselves, were helpful.

“*I also think it’s important for the younger kids to know it wasn’t their fault, [needing] the IV, so if they’re able to incorporate that somehow like, ‘oh, you really helped the doctors and the nurses’ makes them not have the mindset, ‘oh, that was because I did something bad and I deserved it’. More like ‘you helped us and did a really good job*.”

—[Participant 9, Nurse]

In addition, providing further distraction to refocus the child away from the IVI was also mentioned.

“*Because if they now have an IV running or an IV saline lock on their hand, they can also be annoyed at that. So that [the SAR] could distract them from that part*.”

—[Participant 10, Physician]

HCPs also identified additional potential roles for an AI-enhanced SAR in pediatric healthcare settings, which included preparation and education for imaging procedures and overnight stays, child engagement in busy waiting areas, and wayfinding for families.

Introducing a SAR to the ED comes with some potential challenges. HCPs identified two broad areas: the ED environment and limitations of the SAR. Both time and space are limited in the ED, so the SAR would need to be effortlessly incorporated into the workflow, must take up minimal space, require little preparation time, and not limit the movement of HCPs, patients, or family members.

“*I think from the physician and nurse side, it has to be relatively seamless. It can’t be a half an hour we have to push the robot out of the closet and turn it on and it has to load. That’s where you lose time. And because we’re so used to doing IVs without a robot, people will very quickly notice if there’s any significant barriers to making it happen. So I think it’s cool [to have] the opportunity to see how it works and once it’s there, the workflow needs to be as seamless as possible without barriers. Because if you put up barriers, people abandon it very quickly*.”

—[Participant 8, Physician]

“*I think all of this stuff needs to be tailored for emergency, which is unscheduled, everyone’s in a little bit of a shock. Parents out of their normal routine, they might have rushed in, they don’t even have money for parking*.”

—[Participant 3, Physician]

Several HCPs highlighted that the SAR will not be appropriate for certain situations and may even hinder some, for example, a child’s escalating behaviour or a patient rapidly becoming more ill. HCPs were concerned with the SAR’s ability to respond appropriately in those situations and suggested a stop function.

“… *all the noise is escalating everything. I’m a little worried on that, that it might become an extra thing of noise that can’t be stopped. Because sometimes we do have to just stop...the noise and just let the child cry and everybody else stop talking*.”

—[Participant 11, Child Life Specialist]

Additionally, HCPs were concerned about the ability of the SAR to respond to each child’s unique needs in a timely and developmentally appropriate way.

“*That could be a challenge, to be able to talk to a two-year-old and a twelve-year-old using the same device is like using completely different language. The words would have to be different; the body language would have to be different*.”

—[Participant 4, Physician]

“*My concerns are in regard to each child being different. Even learning about what makes one child happy is not necessarily going to make another child happy. So if this isn’t working, we need change. How do we quickly change if it’s not working and recognize that every child is going to be different? That’s the struggle of AI*.”

—[Participant 7, Child Life Specialist]

## 4. Discussion

We have presented the results of the semi-structured qualitative interviews discussing the use of AI-enhanced SARs within children’s healthcare settings. In the interviews, pediatric HCPs of various roles characterized their experience in managing children’s procedural pain and distress during IVI, as well as their perspectives surrounding the use of AI-enhanced SARs within the emergency healthcare setting, specifically identifying implementation benefits and challenges. In this section, we first summarise the findings from the qualitative study and then discuss the implications of those results on the design and implementation decisions for the AI-enhanced SAR.

This co-design study has highlighted several implications for the development of AI-enhanced SARs to support work within the pediatric healthcare environment, both for the specific SAR that is currently being developed as well as for other similar SARs that may be deployed in related contexts in the future. Note that the goal is not for the output of the co-design study to serve directly as a “to-do list” for the system implementation. Co-design should be seen as a continuous collaboration process between the end users and the system designers and developers [16].

### 4.1. Comparison to Previous Literature

To our knowledge, no studies have used a co-design methodology with HCPs to inform the design and development of their SAR. However, three studies have used the co-design process with children and one with caregivers/parents—one of these studies is a parallel codesign study completed by our team with children and caregivers [17,20,23]. Similar to our findings with HCPs, most children and caregivers had a positive response to the SAR, were excited about its inclusion in the ED, and felt that pain and distress in children are important to manage [17,20,23]. In the singular study with caregivers, they reported the challenge of managing their stress in addition to their child’s—this was paralleled by the HCPs in our study who also found it challenging to manage inter-connected child and parent anxiety during the IVI [23]. Movement or animated behaviour was noted as being important by children and similarly by HCPs in our study [17,20]. The caregivers in our parallel study highlighted privacy concerns and trust in the robot technology, this was not mentioned by the HCPs in our study [23]. Finally, the importance of tailoring the SARs behaviour and language to the needs of each child was also reported by caregivers [23]. 

### 4.2. Clinical Implications

Within ever-busy and increasingly resource-strained healthcare settings, there is a need for all newly introduced tools and technology to be helpful without a significant increase in resource burden for their introduction, use training, setup, and maintenance. Prior studies of SARs within the pediatric ED context have shown promise but have been limited by the requirement for persistent staff involvement to program, trouble-shoot, and modulate robotic interactions and responses in real time [14,15]. As such, AI-enhanced SARs are well-positioned to be of increased benefit in pediatric healthcare settings compared to previous iterations; they are autonomously adaptive, thus limiting their resource requirements [30,31]. The AI-enhanced social robotic management of children’s pain and distress during procedures may also reduce the burden on already-strained HCPs, which increases the probability of its uptake in this setting. Additionally, AI-enhanced SARs may be able to facilitate child distraction to expediate procedure time, thus optimizing efficiency to maximize the use of HCPs’ time. However, it is important to acknowledge that the purpose of the SAR is not to replace human involvement in mitigating distress and pain management, but as an additional tool that HCPs may use. 

HCPs identified distinct roles for an SAR during different phases of an IVI, most impactfully during pre- and post-procedure phases when fewer or no clinical team members may be present (i.e., due to other clinical obligations). Similar roles were identified in our parallel codesign study with children and caregivers, in particular, children also emphasized the pre- and post-procedure periods as being most important [23]. In preparation for an IVI, HCPs highlighted the main role of the SAR as providing personalized and responsive emotional support and coping strategies, such as breathing exercises or guided meditation. Such preparation exercises are already bolstered by a significant foundation of evidence for reducing pediatric pain and distress [32,33], and by transitioning this task load from HCPs to an SAR, there is the potential to reduce the medicalization of the procedure via increased distraction as well as decrease the cognitive workload for HCPs. Following an IVI, HCPs emphasized the importance of continued distraction and positive reinforcement by having the SAR remain to support the child. The importance of post-procedure debriefing is well known to be critical in shaping how a child perceives and forms long-term memories of associated pain and distress [34]. Previous studies have shown the positive impact of conversation, including positive talk and reinforcement, on re-shaping children’s pain memories to be more positive [34]. The proposed system would likely be able to remain in the patient’s room longer than the HCP staff post-procedure and could adaptively undertake real-time debriefing conversations with children and families to facilitate positive re-framing and memory formation, thereby lessening long-term procedure-related healthcare anxiety. A previous randomized controlled trial using a humanoid robot (non-AI-enhanced) for distraction during an IVI in the pediatric ED demonstrated a statistically and clinically significant reduction in child distress and parental anxiety, as well as increased satisfaction with IVI [14]. Given its similarities to this system, we expect the proposed enhanced SAR to show similar benefits for children and families.

### 4.3. Research and Technical Implications

Any system used in the children’s healthcare space must, above all, be robust and non-intrusive. For the technical side of the system, this means that it is crucial to prioritise reliable tools and approaches rather than potentially more cutting-edge components, even if this means that the potential flexibility of the system may be limited. Similar facilitators were identified in two scoping reviews of the implementation of social robots, although they were carried out in an older adult population or dealt with the management of mental health concerns [35,36].

In addition to the overall identified need for a robust system, several more specific objectives and constraints can be formulated based on direct insights from HCPs. One important objective pertains to the proposed SAR’s suite of distress-management strategies and tools. HCPs felt that the proposed SAR should be equipped with a diverse range of abilities to suit children’s needs, including encouraging dialogue, positive reinforcement phrases, humour, and cognitive behavioural strategies (e.g., breathing techniques, guided imagery, and meditation). Children and caregivers from our parallel codesign study also reported the importance of these abilities [23]. HCPs also felt that the proposed SAR should allow the user (i.e., child) to choose from a selection of options for distraction. Secondly, the proposed SAR should also have the ability to recognize social signals and generate responsive social signals accordingly, as children manifest their distress in different ways (verbally, physically, and emotionally). A useful and successful AI-enhanced SAR should have the technical capability (image recognition, natural language processing, etc.) as well as knowledge of human actions and emotions to continuously assess the situation and respond in a timely and appropriate manner. Furthermore, the proposed SAR should use these tools to assess the child’s developmental age (which may not match their chronological or physically appearing age) and act to support the child accordingly. 

Broadly speaking, the implementation of an SAR in clinical settings may also be challenged by financial costs, the fragility of technology, hygiene management, and institutional barriers to uptake [35,36]. HCPs in this study noted two key constraints related to the proposed SAR adaptability. First, it must not speak over an HCP while information is being delivered. Second, the proposed SAR must not act inappropriately. This includes both emotional insensitivity (e.g., telling a joke when a child is crying) as well as situations in which the SAR shows a lack of awareness of a clinical deterioration (e.g., seizures, loss of consciousness). On the technical side, mechanisms to support these constraints should be developed. If a critical situation is not deemed appropriate, the system should feature an explicit and easy-to-use “kill switch” to enable a system interruption at any time. Furthermore, the system should include a mechanism for anyone in the room to explicitly choose from among the possible behaviours, overriding the autonomous AI-driven behaviour if necessary. 

### 4.4. Strengths and Limitations

The current co-design study has been conducted with a sample of HCPs employed in two large urban academic centres. As such, the insights are potentially limited in their generalizability to smaller healthcare centres with fewer resources and less staff. Furthermore, this assessment focused on pediatric emergency HCPs, whose opinions may not align with those of practitioners of other specialties or in different healthcare settings. Subsequent iterative needs assessments could be undertaken to confirm and/or modify these conclusions for a more diverse group of end users, including parents and children alongside HCPs, depending on the desired application context. On the other hand, a diverse group of practitioners (i.e., nurses, physicians, and child life specialists) strengthens the applicability of the results to other care contexts where pediatric healthcare is provided. The use of a rigorous coding process, whereby each transcript was coded twice by two independent team members, is an additional strength.

### 4.5. Future Work

The insights from this co-design study and a parallel study in children and caregivers [23] are currently being incorporated into the first prototype of the SAR system. The robot’s repertoire of behaviours is based on those identified by the HCPs within the constraints of the robot platform. The mechanism for choosing among the behaviours will concentrate on the social signals identified as most important by the study participants that can be reliably sensed by the robot. During all development processes, we will keep in mind the overarching requirements identified by the HCPs for robustness and ease of use; the system is being built using robust, off-the-shelf components wherever possible, and the decision-making component will include mechanisms for manual overrides where necessary. Upon the development of an initial prototype, usability testing of the robot will be conducted to inform design iterations. Once the system is complete, the efficacy of the robot in reducing pediatric pain and distress during IVIs will be evaluated via a randomized and controlled clinical trial within the target hospital EDs. In parallel, our multi-disciplinary team, including experts from computer science, AI, social robotics, communications, computational linguistics, indigenous languages, and ethics, are exploring the social and ethical implications of using AI-enhanced SARs in children’s healthcare. Insights therein will be used to determine the most ethically sound methods to communicate the role and capabilities of an SAR to children and their caregivers. Though the initial iteration of this SAR will be developed for use in English, this work will also serve to bolster the adaptability of the SAR to other languages and cultural contexts.

## 5. Conclusions

This work completes the crucial initial step of performing a needs assessment and ideation for the user-centered design of an AI-enhanced SAR for use within the pediatric healthcare setting. The objectives and constraints identified herein are foundational to the subsequent design, prototyping, development, testing, and implementation of this technology. A similar approach can be effectively undertaken to understand the needs of healthcare providers in other care contexts for the development of other useful AI-enhanced SARs. 

## Figures and Tables

**Table 1:** Demographic characteristics of healthcare providers.

Characteristics	Healthcare Providers (n = 11)
Sex, n (%)	
Female	8 (73%)
Male	3 (27%)
Age (years), n (%)	
20 to 29	2 (18%)
30 to 39	3 (28%)
40 to 49	2 (18%)
50 to 59	2 (18%)
60 to 69	2 (18%)
Type of healthcare provider, n (%)	
Physician	4 (36%)
Nurse	5 (45%)
Child life specialist	2 (18%)
Education level, n (%)	
Diploma	1 (9%)
Undergraduate	6 (55%)
Post-graduate	1 (9%)
Graduate School (Master or PhD)	2 (18%)
Professional Degree (Medical Degree)	4 (36%)
Years of experience in healthcare, mean (SD)	21 (12.3)
Years of experience in pediatric emergency medicine, mean (SD)	12.6 (9.45)
Experience with voice assistant (e.g., Siri)	6 (55%)
Experience with smart home (e.g., Google home, Alexa)	3 (27%)
Experience with robot technology	4 (36%)

## Data Availability

The data that support the findings of this study are available from the corresponding author, J.S., upon reasonable request.
